# Educational supervision in internal medicine residency training – a scoping review

**DOI:** 10.1186/s12909-023-04629-y

**Published:** 2023-09-07

**Authors:** Cecilie Normann Birkeli, Camilla Normand, Karin Isaksson Rø, Monika Kvernenes

**Affiliations:** 1 Institute for Studies of the Medical Profession, P.O. Box 1153, Oslo, NO-0107 Norway; 2 Department of Quality and Health Technology, University of Stavanger, Stavanger, Norway; 3 Department of Internal Medicine, Stavanger University Hospital, Stavanger, Norway; 4 Center for Medical Education, Department of Clinical Medicine, Faculty of Medicine, University of Bergen, Bergen, Norway

**Keywords:** Educational supervision, Internal medicine, Postgraduate training, Residents, Medical education

## Abstract

**Background:**

Although supervision is an important part of residency training, its scope and how it relates to other types of support, such as mentoring, precepting and feedback, remain unclear. While clinical supervision consists of ongoing instructions and feedback in the workplace setting, educational supervision is a formalized component of postgraduate medical educational and supports the process that facilitates a trainee’s progression throughout their training. Since medical specialties have different supervisory traditions, this study focuses on educational supervision in internal medicine. Our aim was to investigate what is known about educational supervision practices in internal medicine and the role of educational supervision in supporting residents’ learning.

**Methods:**

We conducted a scoping review of the literature on educational supervision in residency training in internal medicine based on Levac et al.’s modification of Arksey and O’Malley’s six-step framework. The literature search was performed in the following databases: Medline, Embase, Web of Science and the Educational Resources Information Center. In addition, we conducted a handsearch in Medical Teacher and Google Scholar. We followed the PRISMA guidelines for systematic research.

**Results:**

Eighteen of the 3,284 identified articles were included in the analysis. We found few empirical studies describing how educational supervision is conducted and what effect routine educational supervision has on residents’ learning. Our findings suggest that the terminology can be confusing and that educational supervision practices in internal medicine has a weak theoretical foundation.

**Conclusion:**

The distinction between educational supervision and other support structures, such as mentoring and feedback, has not been clearly defined in the research literature. We argue that shared terminology is needed to better understand current educational practices and to facilitate clear communication about how to help residents learn.

**Supplementary Information:**

The online version contains supplementary material available at 10.1186/s12909-023-04629-y.

## Introduction

In postgraduate medical education, there has been an increased focus on supervision [1, 2], which is highlighted as an important structure ‘to ensure patient safety and promote professional development’ [1]. While definitions of the term supervision vary with contexts and agendas [3], the most-sited definition put forward by Kilminister [4] describes supervision as:

the provision of monitoring, guidance and feedback on matters of personal, professional and educational development in the context of the doctor’s care of patients. This would include the ability to anticipate a doctor’s strengths and weaknesses in particular clinical situations in order to maximize patient safety. (p. 828)

Several different types of supervision are described in the literature. Educational supervision (ES) is defined as ‘regular supervision taking place in the context of a recognized training programme in order to determine learning needs and review progress’ [5]. Educational supervisors are expected to facilitate reflection, help identify learning needs, plan learning activities and formatively assess residents’ performance [3]. This supports trainees’ progress throughout their training by establishing a trusting environment, providing feedback, using direct observation of clinical practice and planning structured, timetabled sessions [2]. Educational supervisors should provide educational, professional and personal support in a longitudinal commitment that can help residents develop resilience [4, 6, 7].

Another common type of supervision is clinical supervision (CS), which describes the supervising day-to-day management of clinical cases and issues arising from such cases [3]. CS varies from discussions on ward rounds or clinics to more extended and reflective case-based discussions, but it is suggested to be evaluative in nature and closely linked to feedback on performance [8].

CS and ES share many of the same functions; Kilminster’s definition of supervision [4] has been used to define both CS and ES in earlier studies [2, 9]. Whereas CS is fairly well established as part of a longstanding apprenticeship model of residency training, ES was first formally introduced with postgraduate reforms in the 1980 and 1990s [10, 11]. Due to the increased focus on supervision, a guide for effective ES and CS was developed [1, 3]. However, previous studies have found that supervision practices are variable, and that supervisors and trainees can have different perceptions about how much supervision is required and how it should be approached [12].

Since then, a number of other support structures for residents’ learning, such as mentoring programmes [13] and coaching [14], have surfaced. The literature asserts that ES contains elements of mentoring, usually understood as guidance and support from a more experienced colleague, and coaching, which is commonly understood as helping people reach their potential [3]. While mentoring can be informal in being arisen naturally between colleagues or as a part of a formal scheme, coaching can be seen as a form of supervision that does not involve management or assessment [3]. Passi [5] claims that ‘educational supervision offers the unique opportunity to be an effective mentor to the student. The mentoring can be informal or more formal within the scheduled supervising meetings’ (p. 195). Patel [2] argues that although mentoring is not the same as ES, it should be a part of ES. Consequently, many of these educational interventions overlap in their definitions and operation in practice, which leads to confusion about what ES actually is.

Overall, there is limited research on ES in postgraduate medical training [2]. In some fields, such as psychiatry, there has been a longstanding therapeutic tradition, drawing on experiential and social learning theories [15, 16], whereas in somatic medicine ES has received less attention. Therefore, we conducted a scoping review [17] of the literature to explore how ES in specialist training for doctors in somatic medicine is carried out in daily hospital practice. A branch of somatic medicine was selected to narrow down the literature search, and internal medicine was specifically selected because it is a large branch of somatic medicine with distinct supervisory traditions. Our point of departure is that a shared understanding of terms is needed to better understand current educational practices and facilitate clear communication about how to help residents learn.

## Methods

We conducted a scoping review based on Levac et al.’s modification of Arksey and O’Malley’s six-step framework [18, 19]. Scoping reviews are considered appropriate for informing practices, exploring how research is conducted in a given field, providing a foundation for developing additional research or related projects and addressing knowledge gaps. A systematic search in relevant databases was conducted to obtain an overview of research in this field. Studies were selected in line with the predetermined inclusion and exclusion criteria that are described in detail below.

### Identifying the research question

The first step was to formulate the research question and develop a clearly articulated scope of inquiry [17]. Scoping reviews allow for more general exploration of selected literature, have a broad conceptual range and can be appropriate when the literature is vast and complex [17, 20]. Our overall research question for this scoping review is: What is known about ES practice in internal medicine training. In answering this we will focus on definitions of terms, descriptions on how ES is organized and findings on how ES supports learning and empirical evidence.

### Identifying relevant studies

Guided by librarians from the University of Bergen, three of the authors (CNB, MK and CN) determined the inclusion criteria and a search strategy (see Appendix 1) using a Population, Intervention, Comparison, Outcomes and Study (PICOS) design (see Table 1). We piloted the search strategy to get an impression of which search terms yielded relevant hits. The search was conducted in April and Mai 2022 in the following databases: Medline, Embase, Web of Science and the Educational Resources Information Center (ERIC). By using internal medicine as an inclusion criterion to refine the search, we found through random sampling that it could exclude potentially relevant articles that dealt with ES but in which internal medicine was not mentioned or in which internal medicine was only one of many specialties considered. Therefore, we ran a separate search for systematic review articles published from 2015 to April 2022 (not limited to internal medicine) to compensate for this. We also conducted a handsearch to include all volumes of Medical Teacher in the past five years that were not covered in the Ovid databases. In addition, we performed handsearch in Google Scholar for ‘educational supervision’ in ‘internal medicine’ and ‘educational supervision’ in ‘core medical training’.

**Table 1:** Population, Intervention, Comparison, Outcomes and Study (PICOS) design

PICOS	Inclusion criteria	Exclusion criteria
Population	Internal medicine residents (including cardiology), endocrinology, gastroenterology, haematology, infectious diseases, nephrology (renal disease) and respiratory medicine	PhD programmes or supervision, no specialty chosen yet, internship in internal medicine
Intervention	Educational supervision, guidance, mentoring, advisership, conversations and dialogue	
Comparison	Definitions of educational supervision, feedback and support structures that fell under or were similar to educational supervision	
Outcomes	Theoretical foundation of supervision, workplace learning, professional development, professional competence, role modelling, feedback, support, communication, teaching and learning	
Study	All types of scientific papers, articles and study designs were included (longitudinal studies, cross-sectional studies, case studies, observational studies, randomised controlled trials, cohort studies and descriptive studies)	Non-English language articles

### Selecting studies to be included in the review

**Fig. 1:**
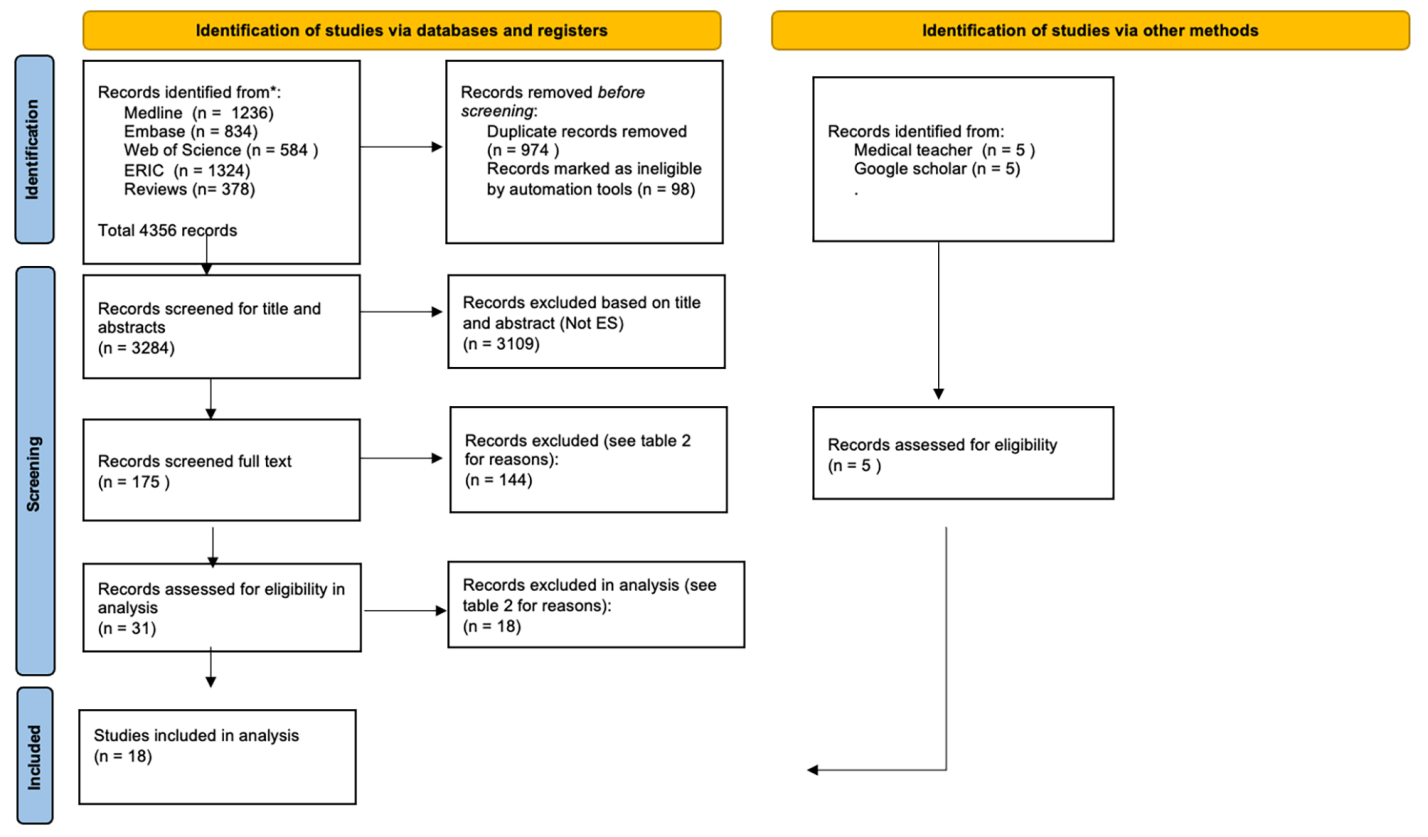
PRISMA flow chart

We followed PRISMA guidelines for systematic research to document the screening process [21]. We used EndNote version 20 and Rayyan (intelligent systematic review) to systematise our data during the screening process, as illustrated in the PRISMA flow chart (Fig. 1). All articles were screened based on title and abstracts using the specified inclusion and exclusion criteria.

CNB screened all 3,284 articles, and the other three authors (KIR, CN, MK) screened a third each, thus ensuring a double-blind review. The resulting 180 articles were full-text screened by MK and CNB, blinded. Conflicts between the two authors’ assessments were resolved through discussions amongst the authors.

We included publications that described a support structure that was formalised, longitudinal and aimed to facilitate reflection on professional and personal development learning needs and provide pastoral support. These inclusion criteria are in line with the definition of ES put forward by Passi [5]. We excluded publications that described CS (understood as observations, feedback and daily support in clinical practice), formative assessment practices and studies on mentoring – which is often associated with reciprocal, voluntary and confidential relationships between a younger and a more experienced colleague – that typically focus on career enhancement, work-life balance and professional identity formation [3].

Importantly, we did not include or exclude articles solely based on terminology. We reviewed all the articles with the intention of identifying educational support structures that fell under the definition of ES. The process of screening was challenged by the overlapping definitions of ES and other support structures such as mentoring, CS and coaching, particularly in cases in which definitions of supervision/mentoring were not offered in the abstracts or in the full text. Full-text articles were excluded based on eight exclusion criteria (see Table 2).

**Table 2:** Reasons for exclusion during full-text screening

Not consistent with educational supervision	65
Different outcome	23
Full-text paper not published	14
Different population	24
Different topic	16
Different study design	10
Different publication type	7
Foreign language	3

### Data characterisation and analysis

From the articles included in the review, we extracted information about year of publication, demographics, study population, publication type, research method and design. Three of the authors (KIR, MK and CNB) extracted information about what terms and definitions were used to describe ES and, if available, what educational theories the publications referred to. Finally, we obtained information about how educational supervision was conducted and organised during training and what evidence was presented to describe how ES supports learning.

### Collating, summarising and reporting the results

The results and analysis are presented below.

## Results

Of the 3284 articles screened, we included 18 studies published between 1996 and 2022, in our data analysis. Most of the articles came from the United States (n = 7); the rest were from the United Kingdom (n = 4), Australia (n = 1), Canada (n = 1), Japan (n = 1), Norway (n = 1), Singapore (n = 1) and Spain (n = 1), as well as a crossover study from the Netherlands, the United States and Canada (n = 1).

### Study characteristics, demographics and population

The articles varied in study design (see Table 3): eighteen were empirical research studies, seven were qualitative studies, six were quantitative studies and four were mixed methods studies.

**Table 3:** Study characteristics, demographics, population, organizing and support

	Reference	Country/study design	Method/data sources	Sample/specialty	Terminology & focus	Descriptions on how ES is organized	Findings on how ES supports learning and empirical evidence
1	Panayiotou & Fotherby (1996) [22]	UK Quantitative	Descriptive statistics Survey	64 house officers, 18 registrars General medicine, geriatric medicine and integrated medicine	**Educational supervision**	“An educational supervisor will be required by every trainee to undertake a formal appraisal of performance and identify short-falls in training.”	The findings show that most doctors considered training and educational task comprised only a minor part of their work. The educational value of clinical activities is redused if supervision, teaching and instruction by more senior staff is lacking. Survey uncovered inadequate supervision in many cases, particularly in out-patients departments.
2	Lycke et al. (1998) [23]	Norway Quantitative	Descriptive statistics Two surveys with four years apart	1991: 88% of 215 invited supervisors 1995: 455 supervisors responded All specialities	**Educational supervision**, Evaluating the effect and impact of a training program in educational supervision	The supervisor and trainee meet regularly to address the trainee’s individual learning needs as they emerge through his work on the wards, self-studies or future challenges.	40% of the participants in the 1995 study indicate that the course had helped them to get started as a supervisor, and 84% indicated that the course had made them better supervisors. In the 1991 study: “Supervisor ranked the role as advocate (30%), promoting professional judgement (26%) and encouraging reflection on actions (17%) as their most important functions.” .
3	Tasker et al. (2014) [7]	UK Quantitative	Descriptive statistics Electronic survey	456 Core Medical Training (CMT) trainees (1 year) 392 CMT trainees 2 year) Internal medicine	**Educational supervision** Measuring number of educational meetings held per year	Gives information on the structure and usefulness of educational supervision the number of educational meetings held per year (median number of meetings was four), the length of the meetings and length of attachment to a single supervisor.	This study identified fundamental problems with the CMT programme such as lack of continuity in supervision and training, overwhelming service provision at the expense of training time and lack of preparation for the role of a medical registrar. A high proportion of trainees did not receive feedback from consultants and many trainees felt that educational supervision was limited.
4	Webb et al. (2017) [25]	UK Mixed methods	Telephone interviews with thematic analysis. Qualitative and quantitative questionnaire	Educational supervisors: 11 semi structured interviews, 191 respondents to questionnaire	**Educational supervision** Formative evaluation of the Educational supervision agreement (EdSA) after 1 year.	Describe that… “In signing the Agreement, the Educational Supervisors commits to undertaking a minimum of 8 hours of continuing professional development (CPD) per year that maps to at least two of the seven framework areas adopted by the General Medical Council and to cover all areas within a 5-year cycle”.	The authors concluded “The evaluation shows the positive expectations of the agreement to deliver on its principles, providing the quasi-regulatory authority to support, recognise and monitor the role of educational supervisors in practise. These findings provide positive reinforcement for the development and roll-out of EdSa and its potential extension to Named Clinical Supervisor.”
5	Diaz et al. (2019) [26]	Spain Quantitative	Descriptive statistics Survey	110 mentors Internal medicine	**Mentoring**	One mentor supervises 5 or fewer residents, 50% report being unable to fully dedicate, 30% of mentors have no training. Most mentors used resident interviews as an approach and they discuss rotations: “Although unifying the amount of time that resident spend working in outpatient’s clinics would be ideal, in a third of the cases there is no supervision”	Mentoring in internal medicine is of good quality but can be improved in 2 key aspects. Supervision and scheduling the rotations calendar adapted for each resident. The use of interviews as a method for detecting problems is widely used, and those centers that do not conduct them should consider implementing them.”
6	Ong et al. (2018) [27]	UK Mixed methods	Statistical analyses, qualitative data was “grouped into categories” qualitative and quantitative questionnaire	110 trainees	**Mentoring** Determining quantitatively if a positive association exists between the mentoring of junior doctors and better training outcomes	Two groups of junior and medical doctors in training were studied and compared targeted training outcomes in a group of trainees who had received mentorship in a structured mentor program versus non-mentored group. Mentored trainees reported higher pass rates and higher confidence and career progression versus non-mentored trainees.	The study showed a positive association between mentoring of junior doctors and better training outcomes. Those who had follow the program had better pass-rate and showed better progression, confidence and reported mentoring in a positive manner.
7	Ramanan et al. (2006) [28]	USA Quantitative	Various statistical analyses survey	329 interns and residents Internal medicine	**Mentoring** Exploring mentoring relationships	One half of the mentored residents reported that their mentor was assigned to them, either by a residency program director, or through a formal mentorship program. Some residents regarded their continuity clinic preceptor as a mentor. Of the remaining mentoring residents, 43% initiated the relationship, 8% described mentoring relationship initiated by a mentor. Most resident met with their mentor every 6 to 12 months, and majority of residents reported this as sufficient.	Findings show that the residents describe a beneficial effect on personal development, receiving helpful advice on career decisions, clinical work and research. A smaller percentage describe assistance finding a position after residency or guidance when facing disappointment or failure.
8	Castiglioni et al. (2004) [29]	USA Quantitative	Univarate statistics, Chi-square, ANNOVA Questionnaire	227 program directors Internal medicine	**Mentoring** Assessing the prevalence of mentoring programs and internal medicine program directors’ attitudes toward mentoring	“49% of the residencies had structured mentoring programs. Programs differed in frequency of meetings, use of evaluations, and presence of curriculum”	**Not indicated**
9	Levy et al. (2004) [30]	USA Mixed Methods	Based on quantitative and qualitative survey data. Discuss experiences on implementing a mentoring program based on earlier reports	Internal medicine	**Mentoring** How to meet key challenges in mentoring relationships between house officers and hospital staff physicians	“Each faculty member was assigned one to three members of the house staff as mentees. Individual relationships last as long as each mentor – mentee pair find their relationship productive.”	“To improve mentoring activities and help identify the qualities of mentoring that are distinct from supervising, precepting or serving as a role model. Top ten attributes are listed on the mentoring web site”. “Both faculty and house staff recognize the value of mentoring to successful career development, but finding time for this important activity is increasingly difficult”
10	Krishna et al. (2019) [31]	Singapore Qualitative	Scoping review, PubMed, Scopus, ERIC, Cochrane	34 articles on role modelling; 9 on teaching & tutoring; 43 articles on coaching; 18 articles on supervision Internal medicine	**Mentoring** and overlapping educational roles (role modelling, teaching and tutoring, **supervision** and coaching)	There must be an balance between individualization of mentoring relationships that includes catering to the mentee’s needs, abilities, goals and situation and ensuring a consistent mentoring approach that is both compliant to prevailing codes of conduct and sufficiently structured to allow effective, timely appropriate, personalized, specific, holistic, longitudinal and accessible evaluations and support for the mentee, mentoring and the mentor relationship.	Mentoring will build better educational interactions. The need for a safe and nurturing working environment that will nurture trusting and enduring mentoring relationships that will not only enhance better role modelling when the mentee had established ties with the mentor but also facilitate discussions that extend beyond professional issues which allow the provision of holistic support. The need for an open and safe mentoring culture that allows open discussions, constructive feedback and frank discussion.
11	Huffman el.al (2021) [32]	USA Qualitative	Constructivist grounded theory semi-structured interviews	15 residents internal medicine	**Feedback conversation**, Characterize residents’ experiences with the tension between feedback as a bidirectional learning conversation and assessment	The authors conceptualised .”that formal assessment are comprised of two roles: the learner as a performer and the supervisors as an audience to the performance by the learner**”**	Study finds that “the involvement over time and the demonstration of investment in the learners’ growth supports a growth mindset and an openness to displaying uncertainty, asking questions, and seeing weaknesses as opportunities for growth.”
12	Sargeant et al. (2017) [33]	Canada, Qualitative	Template analyses and thematic analyses interviews and transcript of feedback sessions	7 residents, 5 supervisors internal medicine, pediatric	**Feedback**, Exploring how residents engage with and use feedback. R2C2 (relationship, reaction, content, coaching)	By using the R2C2 feedback model the authors studied a small group of supervisors consisting of four phases: rapport and relationship building, exploring reactions to feedback, exploring feedback content, coaching for change	This study finds that “residents’ and supervisors’ consistency in reporting being engaged in the feedback discussions.” “Supervisors and residents reported that using the R2C2 model enabled meaningful, collaborative, goal-oriented feedback discussions.”
13	Sargeant et al. (2018) [34]	Canada, USA, Netherlands Qualitative, case study	Realist evaluation of an intervention Debrief interviews, Audiotapes from feedback sessions and learning change plans	45 residents and 21 supervisors from five residency programs family medicine, psychiatry, internal medicine, surgery, anaesthesia	**Feedback and Coaching** Exploring the effectiveness of a feedback model based on relationship building, exploring reactions to the feedback, exploring understanding of feedback content, and coaching for performance change (R2C2)	RC2C – model: relations, reactions, content, change. Program directors engage in reflective feedback conversations about the resident’s assessment data and use the data to plan improvement.	By helping residents engage in feedback conversations and using the information to plan actions. This supportive process is described as coaching
14	Rhodes et al. (2022) [35]	Australia Qualitative	Thematic analysis semi-structured interviews	13 trainees Geriatric medicine	**Consultants/supervisors;** Examine trainees’ experience of postgraduate training,	“Trainees are assessed formally with a summative supervisor’s report completed for each rotation they undertake”	“Findings highlight the variability in clinical learning experiences and opportunities and supervision approaches, (…) The imbalance power between the trainee and the supervisor and feedback operating in one direction (…) Participants consistently expresses a need for more constructive feedback from supervisors, particularly related to areas from improvement, so that they could gauge how they were performing”
15	Tsukube et al. (2020) [36]	Japan Quantitative	Statistical analyses, Retrospective questionnaire survey	179 MDs Surgeons and physicians	**Supervision** Effect of **cognitive apprenticeship** on junior doctors’ perceived professional growth	**Not indicated**	“This study confirmed that the six dimensions of cognitive apprenticeship enhanced the perceived growth of junior doctors, regardless of their clinical domain or period of supervision. (…) Junior physicians tended to receive more coaching, scaffolding and more opportunities for articulation and reflection from their supervisor than junior surgeons, suggesting that the workplace domain influences characteristics of cognitive apprenticeship.”
16	Woods et al. (2010) [37]	USA Qualitative	“Identified themes” focus group, initial focus group follow up (residents and chef residents)	10 faculty members Separate focus group interview with residents and chef residents (N not specified) Internal medicine	**Postgraduate Medical** **Education Advisor** Clarify the role of resident advisors and mentors	“A mentor is often selected to match the resources and expertise with a residents’ needs or professional interests. An advisor is assigned with a role to councel and guide the resident through the residency process, procedures, and key learning milestones.” “Residents usually have 1 advisor during training but could have multiple mentors”	The program leaders in this study synthesized a coherent job description of the program advisor based on identified roles as follows: listener, goal setter, residency planner, evaluator and problem solver, collaborator, and scholar. As an outcome of this process, Duke University has created an advisor toolkit which clearly defines the advisor role, advisor expectations, guidelines for advising, vital resources for advising, required checklist and professional development tools.
17	Patel et al. (2020) [38]	USA Program evaluation. mixed methods	Descriptive statistics, identification of overarching themes, qualitative and quantitative survey, focus group interviews with faculty	50 residents, 21 faculty internal medicine	**Guidance** Describe the implementation of a “mentoring, advising, and/or coaching” (MAC) program	Describing MAC program with goals on identify trainee needs in achieving personal and career goals and pair trainees with faculty who could help them attain these goals in an individualized manner. “Meetings focused on issues mentees wished to discuss at that time, regardless of the outlined roadmap.”	Findings in this study showed that “Residents acknowledged the importance of having a MAC-faculty who was not evaluating them, and with whom all discussions would be confidential. MAC faculty provided emotional support and helped residents navigate obstacles, in addition to understand their individual motives.” “Given the individual needs of each mentee at different points of training, it was felt that mentoring, advising, and coaching were all helpful and should remain in the scope of the program”
18	Ramani et al. (2020) [39]	USA Qualitative	Thematic analysis observations of feedback conversation, interviews	6 faulty preceptors, 12 residents internal medicine	**Precepting** Exploring residents’ perceptions of content and impact of their feedback conversations in preceptor dyads	“The internal medicine training program comprises approximately 150 residents, who are assigned a continuity clinic preceptor for the duration of their training. The program communicates expectations for regular feedback conversations, but no specific frequency or structure is recommended”	“Residents reported that their comfort in discussing challenges and receptivity to constructive feedback resulted from collegial longitudinal relationships with preceptors, trust in their judgement, and conviction about faculty investment in their growth.” “Although some preceptors appeared comfortable in guiding residents’ formulation of next step, others provided vague action plans that left residents uncertain about the plan and the follow-up.”

### Definitions of terms and theoretical anchoring

All the included studies defined the terms used or provided descriptions of the support structures. Four of the articles used the term ES (see Table 3) In one of these [22], an ‘educational supervisor’ is described as a senior doctor formally appointed for each trainee, who is expected to engage in formal appraisal exercises at regular intervals, provide feedback to trainees and identify shortfalls in training.

According to another study [23]:

The underlying idea of educational supervision is to support the professional development of trainees as they progress through the specialist training programme. Supervision aims to strengthen the institutional affiliation of each trainee, further the trainee’s reflection on theory and practice in their discipline and engage the trainee in ethical considerations. (p. 337)

This was the only study that drew on a theoretical framework to explain the purpose of ES – with reference to Schön [24] and ‘the reflective practitioner’ – framing ES as a practice that aims to facilitate reflection.

Instead of defining ES as such, a third study [25] described the responsibilities of an educational supervisor as follows:

Educational supervisors must provide evidence of suitable attitudes and behaviours (for example, from multisource feedback, evaluations of teaching, placement feedback forms, General Medical Council survey results, trainee audits and analysis of critical incidents) which is reviewed in an annual appraisal of the trainer role. (p. 2)

One study [7] did not include a description or definition of ES, but its authors used the term and added empirical knowledge on ES practices through analyses of quantitative data.

**Fig. 2:**
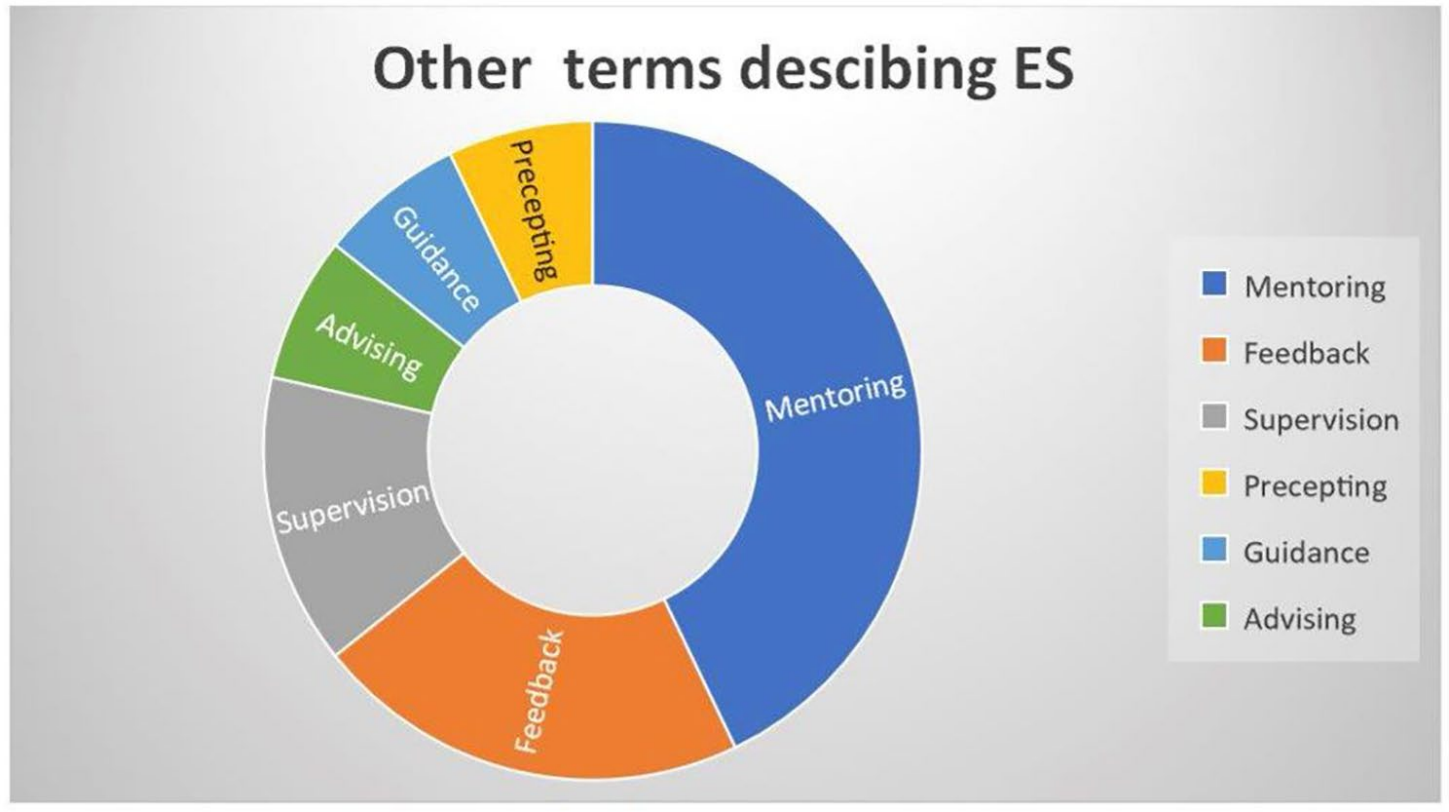
Illustration of other terms used in 14 studies published between 1996 and 2022 to describe practices that fell under the definition of ES

The remaining 14 studies described support practices in line with the definition of ES but used other terms (see Table 3). The most common term was ‘mentoring’, which was used in six studies [26–31], followed by ‘feedback’, which was described in three studies [32–34], ‘supervision’ in two studies [35, 36], ‘advising’ [37], ‘guidance’ [38] and ‘precepting’ [39] in one study each. Figure 2 illustrates the variations in the terms used to describe ES in our material.

### How ES is structured and conducted at work

Seventeen studies provide descriptions or findings on how ES practices were organised (see Table 3). Fourteen studies described time spent on ES practises (see Table 4).

**Table 4:** Studies describing time, duration, and background of supervisors

Reference:	Meetings/conversations occurs regularly or are scheduled:		Time/Duration:		By whom:
	Based on reports of existing practice	Based on reports from intervention (I) or programme (P), evaluation of a model (EM) or a programme (EP), or implementing a programme (IP)	Frequency held per year (Y) or per month (M) or per week (W)	Lasting approximately 1 h	
Panayiotou & Fotherby (1996) [22]	X		W	X	Consultant
Lycke et al. (1998) [23]		EP			Consultant
Tasker et al. (2014) [7]	X		Y	X	Medical consultant
Webb et al. (2017) [25]	X		W	X	Clinicians
Diaz et al. (2019) [26]	X				Internal medicine mentors
Ong et al. (2018) [27]		I			Senior registrars and consultants
Ramanan et al. (2006) [28]		P	M		
Castiglioni et al. (2004) [29]		P			
Levy et al. (2004) [30]	X		Y		Internal medicine faculty members
Sargeant et al. (2017) [33]		EM			
Sargeant et al. (2018) [34]		EM			
Rhodes et al. (2022) [35]	X				Summative supervisors report
Patel et al. (2020) [38]		IP	Y		Internal medicine faculty
Ramani et al. (2020) [39]	X				6 faculty preceptors

Some of these studies reported on how the organization of ES occurred in practise. One study [7] showed that 6% of core medical training (CMT) trainees have ES meetings lasting less than 10 min, 48% reported meetings lasting 10–20 min and 46% more than 20 min. Furthermore, the length of attachment to a single educational supervisor was 12 months for 45% of trainees, 4 months for 23%, 24 months for 21% and 6 months for 7%. Another study found that most doctors considered training and educational task only a minor part of their work [22]. A study of medical training for doctors in Norway [23] found that training and career questions were discussed in 55% of the supervisory meetings, medical questions in 23% and job performance in 13% of the meetings. The supervisors ranked their most important roles as advocates (30%), promoting professional judgement (26%) and encouraging reflection on actions (17%). Another study revealed that mentors are responsible for supervising 5 or fewer residents, with 50% reporting inadequate dedication and 30% lacking formal training [26]. One study [28] reported that most residents met with their mentor every 6 to 12 months reported as sufficient by the majority of residents.

One study described implementation and evaluation of a novel mentor, advisor, and coach (MAC) program created for residents, showing that individualized relationships and meeting content were key to success of the program [38]. One study reported that the internal medicine training program included 150 residents with assigned preceptor, and while expectation for regular feedback conversation is communicated, there were no specific structure or frequency recommended [39]. Two studies presented findings on engagement in reflective feedback based on a structured model for feedback and coaching (R2C2) showing that it enabled meaningful and productive feedback conversations [33, 34].

Additional three studies discussed the various roles attached to being an educational supervisor [31, 32, 37]: One described overlapping educational roles and the importance of balancing individualised mentoring needs with consistent mentoring approaches [31]. Another study argued that since residents are constantly engaged in clinical work, they are frequently engaged in feedback conversations with supervising physicians as a part of their daily routines [32]. Finally, the differences between advising and mentoring are described, suggesting that residents usually had one advisor during training but could have multiple mentors [37].

### How educational supervision supports learning and the empirical evidence of educational supervision

Seventeen studies included in our analysis presented empirical evidence addressing how ES supports residents’ learning (see Table 3). Twelve studies focused on the supervisor role and found that involvement over time, investment in learners’ growth, the value of confidential discussions, successful career development, growth of junior doctors, holistic support, and responsibilities in feedback settings, were important [23, 26–28, 30–34, 36–38]. Five studies identified inadequate supervision, such as a power imbalance between the supervisor and trainee, residents not receiving support or weak support such as vague action plans, a lack of continuity in the programme, a lack of investment in learners’ growth and failure to improve supervision practices [7, 22, 32, 35, 39].

Among the studies that used ES terminology, one described the need to implement ES due to inadequate supervision practices [22]. More specifically, the authors called for more structured training and supervision to help residents in their professional and personal development. With reference to the 1993 Calman proposals in which a new specialist grade was suggested, the authors found it worrisome that most doctors considered training only a minor part of their work.

One of the other studies using ES terminology [23] report changes in educational support structures such as better established residency committees, more extensive use of formal education plans and formal appointment of supervisors for trainees, following a systematic supervisory training program. The authors suggest that extensive faculty development for supervisors might have contributed to these changes.

A study conducted in the United Kingdom [7] reported that many trainees felt that ES was unsatisfactory, the authors also pointed out the lack of specific national guidelines in the United Kingdom for the optimal duration of an educational supervisor meeting.

Another study [25] showed that most of the educational supervisors participating in this study believed that a three-party agreement between educational supervisors’, local education providers and the Wales Deanery would help professionalise the ES supervisor role and increase supervisors’ accountability. The authors also suggested providing supervisors leverage to negotiate time for supporting professional activities (SPA) and continuing professional development. The results showed that 63% of respondents agreed or strongly agreed that the EdSA would ensure the quality of ES and indicated an expectation that the agreement would promote and enhance the standards of postgraduate medical education and training.

## Discussion

The 18 articles included in our analyses, offered definitions and descriptions of ES with limited theoretical frameworks. Seventeen studies provided descriptions of how ES is structured and conducted at work and included description of individualized mentoring, regular feedback conversations, different roles on supervisors and varying structures or length of time spent on ES. Furthermore, seventeen studies showed how ES supports learning and the importance of supervisors’ involvement, investment in learners’ growth, confidential discussions and successful career development. However, inadequate supervision, power imbalance and challenges such as unsatisfactory ES and lack of national guidelines were also identified.

Although there is agreement in the literature that ES is an important part of residency training, we found that this was not reflected in the research on internal medicine supervisory practices. Interestingly, the term ‘educational supervision’ was only used in four publications: two from the 1990s, one from 2014 and one from 2017. Only one of these articles drew on a theoretical framework to define ES [23]. A study from 2016 [2] aligns with our impression that there are few studies of ES in internal medicine. Furthermore, approaches to ES remains highly variable and no studies included direct observations of ES sessions. However, we found several articles describing overlapping practices, such as mentoring, feedback and precepting, that fell under the definition of ES. Many of the studies provided incomplete descriptions of how ES is conducted at work. In particular, ES and mentoring seem to share many characteristics, which might imply that the choice of terminology is primarily a result of local traditions or trends.

As Launer [3] points out, ‘terminology can be confusing, and also varies between countries, but definitions are probably less important than understanding the context and purpose of any encounter’ (p. 179). In addition, with the implementation of competency-based medical education (CBME), the increased emphasis on formalised assessments, structured feedback, entrustment ratings and documentation of learners’ progress has led to the questioning of the relationship between supervision, feedback and assessment [40]. This might have contributed to the unclarities of what ES is. It is also important to consider the various supervisory traditions that exist between medical specialities. Whereas procedural-based specialties such as obstetrics and gynaecology and surgery, are more prone to focus feedback on observational tasks, internal medicine relies more on backstage oversight with support from supervisors [41]. Hatala et al. argue that ‘the ward-based system of learning includes regular periods when clinical supervisors are expected to be absent (e.g., nights and weekends). During these times, it is the system that entrusts trainees’ (p. 741). This means that in a hospital setting, CS and ES are performed by various individuals, whereas in specialties such as general practice, the same person can fill both roles [3]. Research from the field within psychiatry where ES has been particularly important has shown significant variations in definitions, roles and expectations of ES [15, 16].

There seem to be differences between the traditions in some European countries and the United Kingdom, where ES is a clearly defined element in residency training. This is also in contrast to the North American countries (US and Canada), where recent trends seem to have brought more focus on assessment into residency training, thus blurring the lines between formative feedback with the purpose of supporting residents and feedback aimed at assessing performance [40]. This can be problematic because the assessment focus, despite being labelled as formative, may supress and replace other support mechanisms that are there to promote learning. Blurring lines between learning and assessment can be problematic, as judgemental and supporting functions are hard to align. Similar tensions might arise if performance- and procedural-based supervision are not sufficiently delineated from the non-judgemental educational support that is integrated in ES. This suggests that there is a need for both CS and ES in the education of internal medicine residents. Trainees and supervisors must also understand the differences between these types of support, especially in work environments in which colleagues and faculty members fulfil many overlapping and sometimes conflicting roles [3]. Similar findings from research within psychiatry supports this impression [16].

The terms dominating the discourse are not without significance. Reflecting on why there are so many partly overlapping and poorly delineated concepts of support, we need to recognize that in comparison to the traditional apprenticeship model, medical education has become increasingly professionalized over the last decades. Formal assessment requirements have been given a key role in the pursuit of better training and thus better patient treatment. One might suggest that we have become alien to a practice that, in fact, has long roots in medical education. Although supervision – clinical and educational – has historically been implicit in the training of new doctors, it now needs to become explicit. We argue that it is time to revitalise ES to better align educational support structures with local needs.

### Implication for future research

The few studies on ES in internal medicine residency indicates that the concept is not widespread or well known. The low search yield illustrates that there is a research gap on how ES practise is carried out in daily practice. There is a need for both theoretical and empirical research to improve our understanding of how internal medicine residents are supported in their training, how it is experienced from the perspective of both supervisors and trainees. We also suggest that similar studies should be carried out in other field of somatic medicine for broader exploration of existing knowledge. Furthermore, observational studies on ES practice that could provide knowledge on the content of ES would probably contribute to a broader understanding of what ES actually is.

### Strengths and limitations of this study

We performed an extensive and broad search and screened publications to identify the practice of ES. Several reviewers conducted blind screening as well as additional searches in Google Scholar and Medical Teacher. We consider it a strength that our search was guided by a definition of ES, allowing us to map supervising practices, regardless of the terminology used.

A weakness of this study is that limiting our search to internal medicine made it difficult to comment on ES of other junior doctors or residency training in other specialties.

## Conclusions

Our findings indicate that there is no universal definition of ES and that it has a weak theoretical foundation in internal medicine residency. There is also a lack of empirical studies exploring how ES is conducted and how the term is understood in relation to overlapping concepts by both trainees and supervisors. In such explorations, explicit definitions of terms and rich descriptions of practices are needed to avoid silos and to enhance knowledge production across terminological cultures and local supervising regimes. Considering the growing need for education of new doctors it is likely that there will be fewer opportunities for spontaneous individual follow-up of learners. We need to further explore the connection between the intention of what ES is supposed to be and knowledge of what it actually is, based on research on ES practises in internal medicine.

### Electronic supplementary material

Below is the link to the electronic supplementary material.

Supplementary Material 1

## Data Availability

All data generated or analysed during this study are included in this article and its supplementary files.

## List of abbreviations

ES: Educational supervision
CS: Clinical supervision
PICOS: Population, Intervention, Comparison, Outcomes and Study
PRISMA: Preferred Reporting Items for Systematic Reviews and Meta-Analyses
