# Supplementary material for: Educational supervision in internal medicine residency training – a scoping review
Source: BMC Med Educ. 2023 Sep 7;23:644. doi: 10.1186/s12909-023-04629-y (PMC10486128; doi:10.1186/s12909-023-04629-y)
Supplement: Supplementary file 1 — Supplementary Material 1 [file 12909_2023_4629_MOESM1_ESM.docx]

**Appendix 1.**

**Search strategy used in databases**

Comments:

/ = subject heading

Ti,ab,kw = search es in title, abstract or author keywords

**Embase (Ovid) <1974 to 2022 April 28>**

1 residency education/ 30739

2 ((resident* or residenc* or "clinical attachment*" or "clinical rotation*" or "clinical placement*" or doctor* or physician* or trainee* or postgraduate* or graduate*) adj6 (training or education* or speciali* or "professional development")).ti,ab,kw. 125803

3 1 or 2 142797

4 (supervision or supervisor* or mentor* or "guidance conversation" or "planned conversation" or feedback or "formative assessment*").ti,ab,kw. 284392

5 3 and 4 15311

6 exp internal medicine/ 301725

7 ("internal medicine" or Cardiology or Endocrinology or Gastroenterology or geriatrics or "geriatric medicine" or Hematology or "Infectious Disease* Medicine" or "Communicable disease* Medicine" or Nephrology or "Renal disease*" or "Respiratory Medicine").ti,ab,kw. 421128

8 6 or 7 637531

9 5 and 8 2034

10 limit 9 to conference abstract 1200

11 9 not 10 834

**Ovid MEDLINE(R) and Epub Ahead of Print, In-Process, In-Data-Review & Other Non-Indexed Citations and Daily <1946 to April 28, 2022>**

1 "Internship and Residency"/ 56848

2 ((resident* or residenc* or "clinical attachment*" or "clinical rotation*" or "clinical placement*" or doctor* or physician* or trainee* or postgraduate* or graduate*) adj6 (training or education* or speciali* or "professional development")).ti,ab,kw. 91833

3 1 or 2 128062

4 (supervision or supervisor* or mentor* or "guidance conversation" or "planned conversation" or feedback or "formative assessment*").ti,ab,kw. 218802

5 3 and 4 11452

6 exp Internal Medicine/ 111615

7 ("internal medicine" or Cardiology or Endocrinology or Gastroenterology or geriatrics or "geriatric medicine" or Hematology or "Infectious Disease* Medicine" or "Communicable disease* Medicine" or Nephrology or "Renal disease*" or "Respiratory Medicine").ti,ab,kw. 227189

8 6 or 7 304168

9 5 and 8 1236

# Web of Science (Clarivate) Indexes=SCI-EXPANDED, SSCI, A&HCI, ESCI Timespan=All years 29. 4.2022

# 1: 73,287 TOPIC: ((resident* or residenc* or internship* or interns or intern or housemanship* or "house staff*" or doctor* or physician* or trainee*) NEAR/5 (training or education* or speciali* or "professional development") )

# 2: 475,172 TOPIC: (supervision or supervisor* or mentor* or "guidance conversation" or
"planned conversation" or feedback or "formative assessment*")

# 3: 7,452 #2 AND #1

# 4: 275,106 TS=("internal medicine" or Cardiology or Endocrinology or Gastroenterology or geriatrics or "geriatric medicine" or Hematology or "Infectious Disease* Medicine" or "Communicable disease* Medicine" or Nephrology or "Renal disease*" or "Respiratory

Medicine")

# 5: 584 #4 AND #3

**Search History**

| **Last Run Via** | Friday, May 06, 2022 1:29:45 PM |
| --- | --- |
| Interface - EBSCOhost Research Databases Search Screen - Advanced Search Database – ERIC  **Date: 2022-05-06** |  |

| **#** | **Query** | **Results** |
| --- | --- | --- |
| S1 | DE "Specialization" | 2,300 |
| S2 | DE "Medical Education" | 9,874 |
| S3 | DE "Professional Development" | 24,807 |
| S4 | DE "Professional Continuing Education" | 5,242 |
| S5 | DE "Graduate Medical Education" | 1,313 |
| S6 | DE "Foreign Medical Graduates" | 119 |
| S7 | DE "Graduates" | 1,501 |
| S8 | DE "Physicians" | 4,329 |
| S9 | DE "Apprenticeships" | 3,541 |
| S10 | S1 OR S2 OR S3 OR S4 OR S5 OR S6 OR S7 OR S8 OR S9 | 48,593 |
| S11 | ( TI ((resident OR residenc* OR "clinical placement" OR "clinical rotation" OR "clinical attachment" OR doctor* OR physician* OR trainee* OR postgraduate* OR graduate* OR apprentice*) N5 (training OR education* OR speciali* OR "professional development")) ) OR ( AB ((resident OR residenc* OR "clinical placement" OR "clinical rotation" OR "clinical attachment"OR doctor* OR physician* OR trainee* OR postgraduate* OR graduate* OR apprentice*) N5 (training OR education* OR speciali* OR "professional development")) ) | 26,267 |
| S12 | S10 OR S11 | 69,974 |
| S13 | DE "Supervision" | 4,404 |
| S14 | DE "Practicum Supervision" | 825 |
| S15 | DE "Supervisors" | 2,918 |
| S16 | DE "Mentors" | 15,573 |
| S17 | DE "Supervisor Qualifications" | 342 |
| S18 | DE "Supervisor Supervisee Relationship" | 1,300 |
| S19 | DE "Feedback (Response)" | 16,019 |
| S20 | DE "Guidance" | 2,238 |
| S21 | DE "Formative Evaluation" | 8,794 |
| S22 | DE "Specialists" | 2,788 |
| S23 | S13 OR S14 OR S15 OR S16 OR S17 OR S18 OR S19 OR S20 OR S21 OR S22 | 50,787 |
| S24 | ( TI (supervision OR supervisor* OR mentor* OR "guidance conversation*" OR "planned conversation*" OR feedback OR "formative assessment*" OR "formative evaluation*") ) OR ( AB (supervision OR supervisor* OR mentor* OR "guidance conversation*" OR "planned conversation*" OR feedback OR "formative assessment*" OR "formative evaluation*") ) | 76,873 |
| S25 | S23 OR S24 | 93,766 |
| S26 | DE "Medicine" | 2,376 |
| S27 | DE "Internal Medicine" | 333 |
| S28 | DE "Communicable Diseases" | 1,276 |
| S29 | DE "Geriatrics" | 1,052 |
| S30 | S26 OR S27 OR S28 OR S29 | 4,990 |
| S31 | ( TI (medic* OR clinical OR cardiology OR geriatric* OR endocrinology OR gastroenterology OR hematology OR "infectious disease*" OR "communicable disease*" OR nephrology OR "renal disease*") ) OR ( AB (medic* OR clinical OR cardiology OR geriatric* OR endocrinology OR gastroenterology OR hematology OR "infectious disease*" OR "communicable disease*" OR nephrology OR "renal disease*") ) | 57,517 |
| S32 | S30 OR S31 | 59,378 |
| S33 | S12 AND S25 AND S32 | 1,324 |

**Reviews on supervision and residency, without limiting to internal medicine**

**Embase (Ovid) <1974 to 2022 April 28>**

1 residency education/ 30739

2 ((resident* or residenc* or "clinical attachment*" or "clinical rotation*" or "clinical placement*" or doctor* or physician* or trainee* or postgraduate* or graduate*) adj6 (training or education* or speciali* or "professional development")).ti,ab,kw. 125803

3 1 or 2 142797

4 (supervision or supervisor* or mentor* or "guidance conversation" or "planned conversation" or feedback or "formative assessment*").ti,ab,kw. 284392

5 3 and 4 15311

6 exp internal medicine/ 301725

7 ("internal medicine" or Cardiology or Endocrinology or Gastroenterology or geriatrics or "geriatric medicine" or Hematology or "Infectious Disease* Medicine" or "Communicable disease* Medicine" or Nephrology or "Renal disease*" or "Respiratory Medicine").ti,ab,kw. 421128

8 6 or 7 637531

9 5 and 8 2034

10 limit 9 to conference abstract 1200

11 9 not 10 834

12 limit 5 to "systematic review" 223

13 limit 12 to yr="2015 -Current" 178

**Ovid MEDLINE(R) and Epub Ahead of Print, In-Process, In-Data-Review & Other Non-Indexed Citations and Daily <1946 to April 28, 2022>**

1 "Internship and Residency"/ 56848

2 ((resident* or residenc* or "clinical attachment*" or "clinical rotation*" or "clinical placement*" or doctor* or physician* or trainee* or postgraduate* or graduate*) adj6 (training or education* or speciali* or "professional development")).ti,ab,kw. 91833

3 1 or 2 128062

4 (supervision or supervisor* or mentor* or "guidance conversation" or "planned conversation" or feedback or "formative assessment*").ti,ab,kw. 218802

5 3 and 4 11452

6 exp Internal Medicine/ 111615

7 ("internal medicine" or Cardiology or Endocrinology or Gastroenterology or geriatrics or "geriatric medicine" or Hematology or "Infectious Disease* Medicine" or "Communicable disease* Medicine" or Nephrology or "Renal disease*" or "Respiratory Medicine").ti,ab,kw. 227189

8 6 or 7 304168

9 5 and 8 1236

10 limit 5 to "systematic review" 148

11 limit 10 to yr="2015 -Current" 94

# Web of Science (Clarivate) Indexes=SCI-EXPANDED, SSCI, A&HCI, ESCI Timespan=All years 29. 4.2022

# 1: 73,287 TOPIC: ((resident* or residenc* or internship* or interns or intern or housemanship* or "house staff*" or doctor* or physician* or trainee*) NEAR/5 (training or education* or speciali* or "professional development") )

# 2: 475,172 TOPIC: (supervision or supervisor* or mentor* or "guidance conversation" or
"planned conversation" or feedback or "formative assessment*")

# 3: 7,452 #2 AND #1

# 4: 275,106 TS=("internal medicine" or Cardiology or Endocrinology or Gastroenterology or geriatrics or "geriatric medicine" or Hematology or "Infectious Disease* Medicine" or "Communicable disease* Medicine" or Nephrology or "Renal disease*" or "Respiratory

Medicine")

# 5: 584 #4 AND #3

# 6 #2 AND #1 and Review Articles (Document Types) 421

# 7 #2 AND #1 and Review Articles (Document Types) and 2022 or 2021 or 2020 or 2019 or 2018 or 2017 or 2016 or 2015 (Publication Years) 293

# 8 TI=((systematic or scoping or literature or narrative) ) 607,551

# 9 #7 AND #8 106
